# Haemofiltration in newborns treated with extracorporeal membrane oxygenation: a case-comparison study

**DOI:** 10.1186/cc7771

**Published:** 2009-04-03

**Authors:** Karin Blijdorp, Karlien Cransberg, Enno D Wildschut, Saskia J Gischler, Robert Jan Houmes, Eric D Wolff, Dick Tibboel

**Affiliations:** 1 Department of Intensive Care, Erasmus MC Sophia Children's Hospital, Dr Molewaterplein 60, 3015 GJ Rotterdam, The Netherlands; 2 Department of Pediatric Nephrology, Erasmus MC Sophia Children's Hospital, Dr Molewaterplein 60, 3015 GJ Rotterdam, The Netherlands

## Abstract

**Introduction:**

Extracorporeal membrane oxygenation is a supportive cardiopulmonary bypass technique for patients with acute reversible cardiovascular or respiratory failure. Favourable effects of haemofiltration during cardiopulmonary bypass instigated the use of this technique in infants on extracorporeal membrane oxygenation. The current study aimed at comparing clinical outcomes of newborns on extracorporeal membrane oxygenation with and without continuous haemofiltration.

**Methods:**

Demographic data of newborns treated with haemofiltration during extracorporeal membrane oxygenation were compared with those of patients treated without haemofiltration in a retrospective 1:3 case-comparison study. Primary outcome parameters were time on extracorporeal membrane oxygenation, time until extubation after decannulation, mortality and potential cost reduction. Secondary outcome parameters were total and mean fluid balance, urine output in mL/kg/day, dose of vasopressors, blood products and fluid bolus infusions, serum creatinin, urea and albumin levels.

**Results:**

Fifteen patients with haemofiltration (HF group) were compared with 46 patients without haemofiltration (control group). Time on extracorporeal membrane oxygenation was significantly shorter in the HF group: 98 hours (interquartile range (IQR) = 48 to 187 hours) versus 126 hours (IQR = 24 to 403 hours) in the control group (*P *= 0.02). Time from decannulation until extubation was shorter as well: 2.5 days (IQR = 0 to 6.4 days) versus 4.8 days (IQR = 0 to 121.5 days; *P *= 0.04). The calculated cost reduction was €5000 per extracorporeal membrane oxygenation run. There were no significant differences in mortality. Patients in the HF group needed fewer blood transfusions: 0.9 mL/kg/day (IQR = 0.2 to 2.7 mL/kg/day) versus 1.8 mL/kg/day (IQR = 0.8 to 2.9 mL/kg/day) in the control group (*P*< 0.001). Consequently the number of blood units used was significantly lower in the HF group (*P*< 0.001). There was no significant difference in inotropic support or other fluid resuscitation.

**Conclusions:**

Adding continuous haemofiltration to the extracorporeal membrane oxygenation circuit in newborns improves outcome by significantly reducing time on extracorporeal membrane oxygenation and on mechanical ventilation, because of better fluid management and a possible reduction of capillary leakage syndrome. Fewer blood transfusions are needed. All in all, overall costs per extracorporeal membrane oxygenation run will be lower.

## Introduction

Extracorporeal membrane oxygenation (ECMO) is a supportive cardiopulmonary bypass (CPB) technique for patients with acute reversible cardiovascular or respiratory failure. Many ECMO candidates have an increased inflammatory response with capillary leakage before the start of ECMO because of asphyxia, hypoxia and shock. ECMO treatment in itself will trigger or aggravate a systemic inflammatory response (SIRS), resulting in a so-called capillary leakage syndrome [1]. High levels of circulating endotoxins, exotoxins, interleukins and leukotrienes influence the basal membranes [2]. Moreover the ECMO system activates leucocytes, thrombocytes and the complement system [3,4]. This leads not only to water and small molecule leakage through the capillary membrane, but also to leakage of relatively large molecules, including albumin. Permeation of circulating albumin from the blood compartment into the extracellular space often results in generalised oedema. The blood pressure will fall due to extravasation of water and proteins, necessitating administration of oncotic agents and/or vasopressor drugs. Low blood pressure and tissue oedema will potentially cause deficient tissue perfusion and oxygenation leading to multi-organ failure, of which lung and kidney failure are most prominent.

As early as 20 years ago Zobel and colleagues described that haemofiltration rapidly corrected hypervolaemia and pulmonary oedema in nine critically ill children with multi-organ failure [5]. *In vitro *and *in vivo *studies meanwhile have shown that haemofiltration counteracts SIRS by decreasing inflammatory mediators [6-8].

Later studies focused on haemofiltration as a method of preventing multi-organ failure due to capillary leakage syndrome in children during cardiac surgery on CPB [9]. Journois and colleagues reported that haemofiltration resulted in the removal of water and inflammatory proteins from the blood, and consequently in less pulmonary oedema and improved pulmonary function. Time on mechanical ventilation could therefore be shortened and the postoperative alveolo-arterial oxygen gradient improved [10,11]. Haemofiltration is also associated with faster recovery of left ventricular function of the heart, better diastolic compliance, better contractility and less myocardial oedema as recorded by trans-oesophageal echocardiography during CPB [12,13].

Kelly and colleagues reported that pulmonary oedema increases time on ECMO [14]. The potentially favourable effects of haemofiltration during CPB instigated the use of haemofiltration in infants on ECMO in the Erasmus MC – Sophia Children's Hospital since August 2004. It was intended to prevent and diminish the capillary leakage syndrome, and thus to shorten time on ECMO, time on ventilatory support, to lower numbers of blood transfusions, and consequently to reduce overall mortality and costs in this group.

Therefore, since October 2004 in all patients receiving ECMO a haemofilter was incorporated in the ECMO system independent of kidney function. Initially the haemofilter was incorporated after cannulation due to logistic procedures. The current case-comparison study aimed to evaluate the potential benefit of haemofiltration in ECMO patients by comparing clinical parameters in patients on ECMO with and without continuous haemofiltration.

## Materials and methods

### Setting

The intensive care unit (ICU) of the Erasmus MC-Sophia Children's Hospital, Rotterdam, the Netherlands, is a large tertiary facility. It is one of two designated ECMO centres in the Netherlands with 30 to 40 ECMO runs annually, including newborns and children up to 18 years of age. The referral area for ECMO has eight million inhabitants with about 90,000 newborns annually.

### Study design

This was a retrospective case-comparison study. Demographic data of all newborns (less than 28 days post partum) on ECMO treated with haemofiltration (HF group) between October 2004 and October 2006 were compared with newborns treated without haemofiltration (control group) in the previous two years (October 2002 to October 2004) in a 1:3 case-comparison study. Cases and controls were matched for age, weight, diagnosis and ECMO-mode. Inclusion criteria were: in need of ECMO treatment, younger than 28 days and, in the HF group, with haemofiltration. To evaluate the effects of continuous veno-venous haemofiltration (CVVH) during ECMO versus the control group, only those patients receiving CVVH within three hours after starting ECMO were included. We excluded patients treated with furosemide in the HF group to eliminate possible confounding effects of additional diuretic treatment on fluid management.

Controls consisted of a series of consecutive patients taken from the previous two years who were not treated with haemofiltration. Controls were matched for age, weight, diagnosis and ECMO-mode.

### ECMO, haemofiltration and fluid management

The ECMO circuit was primed with 180 mL of a mixture of packed red blood cells, albumin, 100 mL balanced electrolyte solution saline-adenine-glucose-mannitol and 500 units heparin. The ECMO flow at the start was set between 120 and 150 mL/kg/minute. Post-pump pressure was between 200 and 400 mmHg. The filter (Multiflow 60, Hospal, Lyon, France) was placed parallel to the ECMO circuit, distal to the ECMO roller pump. Pressure was measured proximal and distal to the filter. The pressure difference was kept constant at 40 mmHg.

In the filtration group, the predilution flow rate of the filtration fluid (HF-BIC32, Dirinco, Rosmalen, The Netherlands) was as the default of 50 mL/kg/hour. Transfusions with erythrocytes and platelets were administered isovolaemically by ultrafiltrating as much fluid from the patient as the administered blood product. Ultrafiltration was targeted to achieve a normal or negative fluid balance depending on the clinical condition of the patient while maintaining normal haemodynamic parameters. During SIRS and the resulting capillary leakage syndrome this could not always be achieved. In the control group, patients were treated with either continuous or intermittent furosemide infusions to achieve the above mentioned targets as reported earlier by our group [15]. Transfusion of blood products in this group were performed by isovolaemic exchange with whole blood drawn from the ECMO system in an equal amount to the transfused volume thereby maintaining normal haemodynamic parameters. With some exceptions the primary ECMO mode was veno-arterial.

### Data collection and analysis

The following data were retrieved from our Patient Data Management System: physiological parameters, medication, infusions, urinary output, CVVH, ECMO and ventilator settings, fluid balance, laboratory tests and interventions. These data had been collected every hour. Primary outcome measurements were: time on ECMO in hours, time between decannulation and extubation in days and overall mortality. Secondary outcome parameters were: total and mean fluid balance, urine output in mL/kg/day, total doses of vasopressors, blood products and fluid bolus infusions, serum creatinine, urea and albumin levels, and overall costs. Fluid balance was assessed as mean net fluid balance per ECMO day, by measuring total fluid input and output and dividing the difference by the time on ECMO. The difference between predilution and filtration flow rate was included.

The amount of inotropic support was calculated, as reported previously, by the so-called vasopressor score: (dopamine dose (μg/kg/minute) × 1) + (dobutamin dose (μg/kg/minute) × 1) + (noradrenaline (μg/kg/minute) × 100) + (adrenaline (μg/kg/minute) × 100) [16,17].

### Statistics

All data are presented as median (interquartile range (IQR)) unless indicated otherwise. Differences between the groups were tested for their statistical significance by Mann-Whitney U non-parametric test for unpaired data, the Pearson's chi squared test and the Fisher's exact test, according to the character of the variable. A *P* < 0.05 was considered significant.

### Informed consent

Due to the design of the study consisting of a retrospective case-record evaluation, Institutional Review Board approval and the need for informed consent was waived according to Dutch law.

## Results

### Patient profiles

Fifteen patients with haemofiltration (HF group) were compared with 46 patients without haemofiltration (control group). Patient characteristics are shown in Table 1. Median postpartum age on admission was 2.2 days (IQR = 0.9 to 6.7 days) in the HF group and 1.7 days (IQR = 0.5 to 18 days) in the control group. Median weight was 3.5 kg (IQR = 2.5 to 5 kg) in the HF group and 3.3 kg (IQR = 1.9 to 5 kg) in the control group.

**Table 1:** Patient profiles

		Control group (n = 46)	HF group (n = 15)	Pearson's chi squares test
		n (%)	n (%)	*P* value
Sex	Female	21 (46)	5 (33)	
	Male	25 (54)	10 (67)	0.44


				Mann-Whitney U test
				
Scores	PRISM III	37 (14 to 90)	35 (17 to 51)	0.29
	PELOD	628(492 to 694)	633 (551 to 651)	0.93
	OI	20 (1 to 30)	20 (10 to 20)	0.82
	AaDO2	25 (14 to 39)	20 (14 to 40)	0.18

				Pearson's chi squared test
				
Start ECMO	Oct 2002 to Aug 2004	38 (83)	0(0)	
	Aug 2004 to July 2006	8 (17)	15 (100)	-

Diagnosis	CDH	16 (35)	3 (20)	
	MAS*	16 (35)	5 (33)	
	Respiratory diseases	7 (15)	2(13)	
	Sepsis	4 (9)	2 (14)	
	Idiopathic PPHN	2 (4)	2 (13)	
	Post-cardiac surgery	1 (2)	1 (7)	0.73

Surgery	No surgery	42(91)	12 (80)	
	CDH closure	3 (7)	2 (13)	
	Great vessel surgery	1 (2)	1 (7)	0.24

ECMO mode	Veno-arterial	44 (96)	13 (87)	
	Veno-venous	2 (4)	2 (13)	0.22


		median (min to max)	median (min to max)	

Body weight (kg)		3.3 (1.9 to 5)	3.5 (2.5 to 5)	0.31
Age (days)		1.7 (0.5 to 18)	2.2 (0.9 to 6.7)	0.28

Values are presented as mean (interquartile range). * One patient died on ECMO.

AaDO2 = alveolar-arterial oxygen tension gradient; CDH = congenital diaphragmatic hernia; ECMO = extracorporeal membrane oxygenation; HF = haemofiltration; MAS = meconium aspiration syndrome; OI = Oxygenation Index; PELOD = pediatric logistic organ dysfunction; PPHN = persistent pulmonary hypertension of the neonate; PRISM = Pediatric Risk of Mortality Score.

Pediatric Risk of Mortality Scores (PRISM) III were calculated retrospectively at the time of admission to the ICU. Most patients were cannulated within 24 hours of admission. Pediatric Logistic Organ Dysfunction (PELOD), Oxygenation Index (OI) and Alveolar-arterial Oxygen Gradient (AaDO2) scores were taken within six hours of cannulation. Although there are more patients with congenital diaphragmatic hernia (CDH) in the control group there are no significant differences in PRISM, PELOD, OI and AaDO2 scores reflecting a similar severity of illness before ECMO. CDH and meconium aspiration syndrome were the most frequent indications for ECMO therapy. Other diagnoses were respiratory distress syndrome, viral or bacterial pneumonia, congenital cystic adenomatoid malformation of the lung, persistent pulmonary hypertension, post-cardiac surgery and sepsis.

In both groups, two children with isolated pulmonary disease were treated with veno-venous ECMO. All other patients, 13 (87%) in the HF group and 44 (96%) in the control group, were treated with veno-arterial ECMO. Three patients in the HF group and four patients in the control group underwent surgery during ECMO, that is, closure of a diaphragmatic defect (n = 5), thoracotomy due to congenital cystic adenomatoid malformation of the lung (n = 1) or correction of a transposition of the great vessels (n = 1) for which post-cardiac surgery ECMO was needed. Furosemide was administered to 40 children in the control group.

### Outcome

Patient outcomes are listed in Table 2. Time on ECMO was significantly shorter in the HF group: 98 hours (IQR = 48 to 187 hours) versus 126 hours (IQR = 24 to 403 hours) in the control group (*P* = 0.02). Time from decannulation until extubation was shorter as well, though not significantly: 2.5 days (IQR = 0 to 6.4 days) versus 4.8 days (IQR = 0 to 121.5 days; *P* = 0.04). Mortality rate was similar in both groups, 3 of 15 in the HF group and 7 of 46 in the control group (*P* = 0.61). Fluid balance per day on ECMO was significantly lower in the HF group compared with the control group (*P* < 0.001).

**Table 2:** Patient outcome

	Control group	HF group	Mann-Whitney U-test
	
	Median (min to max)	Median (min to max)	*P* value
Time on ECMO (hours)	126 (24 to 403)	98 (48 to 187)	0.02
Time until extubation after decannulation (days)	4.8 (0 to 121.5)	2.5 (0 to 6.4)	0.04

Fluid balance (mL/kg/day)	40 (-53 to 214)	-29 (-75 to 60)	< 0.001
Urine (mL/kg/day)	121 (28 to 292)	54 (11 to 94)	0.38
Filtration flow rate in (L/kg/day)	-	1.2 (0.6 to 1.4)	-
Filtration flow rate out (L/kg/day)	-	1.3 (0.6 to 1.7)	-
Blood loss (mL/kg/day)	57 (8 to 135)	12 (6 to 30)	< 0.001
Erythrocyte transfusion (mL/kg/day)	39 (0 to 73)	16 (0 to 35)	< 0.001
Platelet transfusion (mL/kg/day)	37 (0 to 65)	29 (11 to 63)	0.11
Colloids (mL/kg/day)	6 (0 to 37)	4 (0 to 30)	0.25
Pasteurised plasma (mL/kg/day)	18 (0 to 98)	20 (2 to 69)	0.79
Units erythrocytes (units/day)	1.8 (0.8 to 2.9)	0.9 (0.2 to 2.7)	< 0.001
Units thrombocytes (units/day)	0.9 (0.2 to 1.9)	0.7 (0.2 to 2)	0.3

Vasopressor score	7 (0 to 56)	5 (0 to 41)	0.83
Furosemide (mg/kg/day)	1.2 (0 to 2.6)	-	-
Bumetanide (mg/kg/day)	0 (0 to 0.27)	-	-

Maximum serum creatinine (μmol/l)	58 (14 to 91)	49 (28 to 105)	0.17
Maximum serum urea (mmol/l)	6 (1 to 42)	4 (2 to 13)	0.01
Minimum serum albumin	21 (2 to 30)	23 (13 to 28)	0.15
Mortality rate	7 (16)*	3 (21)*,**	0.61
Day of death after decannulation	3.4 (0 to 11.4)	1.5 (-0.5 to 6.4)	0.56

Values are presented as mean (interquartile range). * n (%), ** One patient died on ECMO.

ECMO = extracorporeal membrane oxygenation; HF = haemofiltration.

Patients in the HF group needed fewer blood transfusions than controls 0.9 mL/kg/day (IQR = 0.2 to 2.7 mL/kg/day) versus 1.8 mL/kg/day (IQR = 0.8 to 2.9 mL/kg/day; *P* < 0.001). Consequently the number of used blood units was significantly lower in the HF group (*P* < 0.001). No statistically significant difference was observed between the two groups with respect to volume and number of units of platelet and colloid transfusions. Used colloid solutions included fresh frozen plasma, pasteurised plasma solution and human albumin.

Maximal creatinine values were above normal range in both groups, and tended to be lower in the HF group (*P* = 0.17). Maximal urea level was significantly lower in the HF group (*P* = 0.01). No significant difference was noted between the two groups with respect to the lowest albumin value. Doses of vasopressor did not differ significantly between the groups.

### Costs

Although the need for additional support was higher in the initial phase of CVVH on ECMO personnel costs did not differ between both groups. ECMO nurses were continuously available for the priming of the system and integrated the haemofilter in the ECMO circuit. They took care of both the ECMO circuit (with or without haemofilter) and the patient. A median patient in the control group needed 28 hours more on ECMO and 55 hours more on mechanical ventilation. The total costs per day on ECMO, including costs for personnel, materials and overheads, were calculated at €4328.

The mean total costs per day for treatment on an ICU ward with mechanical ventilation in our institution amounted to €1480. A median of an extra 5.4 units of blood were needed per patient in the control group, representing €964. In the HF group extra costs were generated by 1 or 2 filters (€90 each) and a median of one 5 L bag of substitution fluid (€15).

The profit gained by adding haemofiltration to the ECMO circuit thus amounted to more than €5000.

## Discussion

In 2008 Hoover and colleagues showed that the use of CVVH in paediatric patients on ECMO is associated with improved fluid balance and caloric intake and less diuretics than in case-matched ECMO controls [18]. We report the first study in newborns that shows that haemofiltration during ECMO improves clinical outcome. This is expressed by a shorter duration of ECMO treatment, and of mechanical ventilation after ECMO. Moreover, the use of haemofiltration resulted in fewer blood transfusions in this group.

The calculated cost reduction for each haemofiltrated patient was more than €5000. Although adding haemofiltration to an ECMO circuit may result in the need for additional support, in our centre our ECMO staff are trained to manage the CVVH treatment negating the need for additionally trained nursing support. Adding a treatment to an already complex patient may result in treatment errors. This is always an issue in an ICU setting and difficult to express in terms of cost. This said, we did not have any complications in administering CVVH during ECMO in the study.

Capillary leakage syndrome is a frequent complication of CPB and ECMO leading to generalised oedema, hypotension and ultimately multi-organ failure. Several studies have reported that the use of haemofiltration during and after CPB resulted in less oedema and shorter post-operative ventilation [9-13]. Before starting ECMO, many ECMO candidates already have an increased inflammatory response with capillary leakage because of asphyxia, hypoxia and shock. In an effort to maintain a normal blood pressure, patients are treated with inotropic support, but also unfortunately with ample fluid supplementation. This therapy may result in an increase of generalised oedema and subsequently pulmonary oedema. ECMO treatment aggravates this inflammatory syndrome [1].

The higher need for blood transfusions in the control group is most likely to be because of the possibility of isovolaemic transfusion of blood and platelet transfusions via the haemofilter in the HF group. This may in itself have a beneficial effect on multi-organ failure. Bjerke and colleagues reported that restricting blood transfusions in newborns on ECMO decreased the running time of ECMO by 15% [19]. Tran and colleagues studied factors associated with multi-organ failure in patients with critical trauma. One such factor was the number of blood transfusions received [20]. This relation may be due to a nonspecific host response to transfusions, resulting in progressive multi-organ failure. Multi-organ failure score is one of the major predictors of death on the ICU, so blood transfusions contribute to worse clinical outcome. Modern strategies to deplete red cell transfusions of leucocytes may, however, decrease this risk, as recently indicated in critically ill children by Lacroix and colleagues [21]. Nevertheless, restrictive blood transfusion strategy is recommended in children whose condition is stable.

We did not demonstrate a favourable effect of haemofiltration on multi-organ failure or capillary leakage, expressed as better renal function, lower vasopressor score or less need for fluid resuscitation. Creatinine levels were slightly elevated in both groups [22], and tended to be lower in the haemofiltrated group. The slightly lower level of serum creatinine and urea in the filtrated group can, at least partially, be explained by the convective clearing effect of haemofiltration. There was no statistical difference in other volume supplementations or inotropic support. This study was not designed to evaluate the effect of haemofiltration on SIRS. Due to the retrospective nature of our study, levels of inflammatory mediators were obtained from plasma, urine or filtrate were not available.

We did not find a statistically significant change in mortality rate, but patient numbers in this study are too small to draw conclusions on this aspect of the results. The total mortality rate of 10 in a population of 61 patients (16%) is fairly low, in comparison to both the mortality rate of 53 in a population of 188 patients (28%) in the previous 10 years of ECMO treatment and the overall mortality rate of 24% in the Extracorporeal Life Support Organization registry in newborns treated with ECMO for respiratory failure. Addition of haemofiltration increased fluid extraction during ECMO in our study, expressed by a better overall fluid balance, in contrast to treatment with diuretics.

### Limitations of our study

In this case-comparison study patients were matched for most confounding factors. Due to the relatively small sample size it was not possible to perfectly match cases and controls, resulting in a higher percentage of patients with CDH in the control group. We acknowledge that patients with CDH have a higher overall mortality and morbidity, especially compared with patients with meconium aspiration syndrome. This also applies to patients with idiopathic pulmonary hypertension, constituting 13% of the cases. However, no significant differences in baseline characteristics (Table 1) between the groups exists. Both severity of illness expressed by PELOD and PRISM III scores and severity of respiratory failure expressed by OI and AaDO2 did not differ significantly.

Secondly the groups had been treated during different time periods; however, patients in the HF group were treated two years later than patients in the control group. As ECMO haemofiltration was not introduced until August 2004, the HF group in this single-centre, retrospective study consists of only 15 patients. No significant changes in indications for treatment on ECMO took place over the years and patients were treated by the same team without major infrastructural changes in our ECMO setting.

Furthermore, no data were collected to detect a decrease in inflammatory mediators. Therefore, it is not possible to evaluate the potential favourable effects of haemofiltration on SIRS, that is, through a mechanism that lowers the inflammatory mediator response. An ongoing randomised controlled trial in our institution is expected to yield more information to optimise the value of haemofiltration during ECMO.

## Conclusions

Adding continuous haemofiltration to the ECMO circuit in newborns improves short-term outcome by significantly reducing time on ECMO and on mechanical ventilation, and by a possible reduction of SIRS and capillary leakage syndrome. Furthermore, significantly fewer blood transfusions are needed. Haemofiltration during ECMO decreases costs per ECMO run by €5000. Given the fact that 30 patients per year receive ECMO treatment in our institution, a €150,000 cost reduction per year could be accomplished.

## Key messages

• CVVH during ECMO reduces time on ECMO and time to extubation post-ECMO.

• CVVH during ECMO decreased the need for blood transfusions.

• CVVH during ECMO resulted in a €5000 cost reduction for each ECMO run.

## Abbreviations

AaDO2: alveolar-arterial oxygen tension gradient; CDH: congenital diaphragmatic hernia; CPB: cardiopulmonary bypass; CVVH: continuous veno-venous haemofiltration; ECMO: extracorporeal membrane oxygenation; ICU: intensive care unit; IQR: interquartile range; OI: Oxygenation Index; PELOD: pediatric logistic organ dysfunction; PRISM: Pediatric Risk of Mortality Score; SIRS: systemic inflammatory response syndrome.

## Competing interests

The authors declare that they have no competing interests.

## Authors' contributions

KB evaluated the data. KB and KC wrote the manuscript. EDW (Wildschut), SG, EDW (Wolff) and RH were involved with patient management. EDW helped draft the manuscript. DT coordinated the data evaluation and the writing of the manuscript. All authors read the final manuscript.
